# A versatile fluorescence polarization-based deubiquitination assay using an isopeptide bond substrate mimetic (IsoMim)

**DOI:** 10.1016/j.jbc.2025.110342

**Published:** 2025-06-06

**Authors:** Jiatong Zhang, Jed Allen, Stephanie J. Ward, Lodewijk V. Dekker, Ingrid Dreveny

**Affiliations:** Biodiscovery Institute, School of Pharmacy, University of Nottingham, Nottingham, United Kingdom

**Keywords:** ubiquitin-dependent protease, deubiquitylation (deubiquitination), fluorescence anisotropy, high-throughput screening (HTS), proteolytic enzyme, ubiquitin, inhibitor, fluorescence polarization assay

## Abstract

Deubiquitinases (DUBs) play a critical role in the regulation of various cellular processes, such as protein homeostasis and signaling, rendering them attractive drug targets. However, the generation of reagents for measuring DUB activity typically involves several steps and is not straightforward. Here, we report the development and characterization of a novel fluorescent polarization assay using an isopeptide bond substrate mimetic (IsoMim) that can be made recombinantly in high yields. The IsoMim assay was able to discern the differential activity of ubiquitin-specific protease family members (USP4, USP15, USP11, and USP2), the ubiquitin C-terminal hydrolase UCHL3, and the Machado-Joseph Domain deubiquitinase JOSD2. A competition assay format of the assay was developed that discerned differences between the close paralogues USP15, USP4, and USP11 in interacting with mono-ubiquitin, the isopeptide mimetic ubiquitin-GGG, and the C-terminal truncation variant ubiquitin (1–74). Moreover, dose–response curves and associated pIC50 values using the broad-spectrum inhibitor PR-619 confirmed differential inhibition in the low μM range for four tested DUBs. The successful discrimination of DUB activity and inhibition and the easily scalable generation of the substrate make the IsoMim assay method applicable for high-throughput screening (HTS). This was ascertained in a “pseudo HTS screen” for USP4 inhibitors in which PR-619 was successfully identified as a “pseudo hit.” The developed assay provides a valuable tool for probing DUB activity and the identification and characterization of DUB inhibitors and has the potential to accelerate drug discovery efforts in this area.

Ubiquitin, a highly conserved protein found in all eukaryotic cells, plays a crucial role in various cellular processes by tagging target proteins for degradation, altering their stability, localization, or function (1). Ubiquitination requires a three-enzyme cascade for conjugation of ubiquitin to a target substrate that concludes with the action of an E3 ligase. Deubiquinases (DUBs) counteract the action of ubiquitin ligases by catalyzing the removal of ubiquitin, thereby modulating protein activity. DUBs are vital regulators of the ubiquitin-proteasome system and, given their critical involvement in various cellular pathways, have emerged as attractive therapeutic targets for the treatment of numerous diseases (2), including cancer, neurodegenerative disorders, and immune-related disorders (3, 4, 5).

In humans, over a hundred DUBs are known, and these comprise seven classes with distinct structures, six of which are cysteine proteases. These include ubiquitin-specific proteases (USPs), ubiquitin carboxyl-terminal hydrolases (UCHs), ovarian tumor domain-containing proteases (OTUs), and Machado-Joseph disease protein domain proteases, or alternatively named Josephins (MJDs), Motif interacting with ubiquitin-containing novel DUB family (MINDYs), and zinc finger containing ubiquitin peptidase 1 (ZUP1) (6, 7). Among these, USPs form the largest and most diverse DUB family. Moreover, several bacterial and viral pathogens generate DUBs as part of their repertoire to evade the host immune response (8).

DUBs generally cleave ubiquitinated substrates by hydrolyzing isopeptide bonds formed between the C-terminal glycine of ubiquitin and an ε-amino group of a lysine residue in the substrate protein, although other linkages are known (9). This complicates the generation of DUB substrates as they are not accessible recombinantly.

To enable the study of DUB activity and to facilitate inhibitor discovery, robust, sensitive, and scalable assays are required (10). A widely used assay for DUB activity employs a 7-amino-4-methylcoumarin (AMC)-conjugated ubiquitin substrate Ub-AMC (11), in which the AMC fluorescence signal is internally quenched by the protein moiety until DUB cleavage alleviates the quenching. Various synthetic routes for the creation of Ub-AMC have been described, including exchanging the C-terminal Gly–Gly residues of ubiquitin with Gly–Gly–AMC in a trypsin-catalyzed transpeptidation reaction, intein-based modification, as well as more recent chemical protein synthesis and semi-synthetic methods (12). Since AMC emits in the blue spectral range and compound interference is a known factor when screens are conducted at these wavelengths, a Rhodamine 110 glycine ubiquitin conjugate was developed, synthesized in a multi-step process based on inteins (13) that emits in the green range of the spectrum. However, reagent yields for Ub-AMC/Ub-Rho110 are typically low, while relatively high concentrations are needed for the assays (14).

As an alternative to fluorescence intensity measurements, FRET assays have been employed (15). A DUB assay substrate for homogeneous time-resolved fluorescence is available that uses an N-terminal Yellow-Fluorescent Protein–ubiquitin fusion with an alanine–cysteine extension for coupling of a terbium chelate (16). In this FRET assay, the long lifetime of terbium allows gating of the emission signal to reduce fluorescence interference. Upon DUB cleavage of the conjugate, the time-resolved FRET from Tb to YFP can no longer occur. This assay was suitable for measuring the activity of UCHL3 and, to a lesser extent, UCHL1 and USP5 (16). Furthermore, differently linked di-ubiquitin-based FRET substrates have been developed based on the Rhodamine-TAMRA or other FRET pairs (17, 18).

Fluorescence polarization (FP) assays have also been employed for measuring DUB activity, including a TAMRA-based ubiquitin derivative in which a TAMRA-conjugated peptide or lysine is linked *via* the epsilon amino group of an N-terminal lysine enzymatically or chemically (19, 20, 21). However, the generation of all these substrates is typically a time-consuming multistep process with moderate yields, which is reflected in the price of commercially available assay reagents.

We recently reported an engineered deubiquitinase substrate mimetic that was instrumental in determining the crystal structure of a USP11 Michaelis-like complex and revealed a structural feature for deubiquitination selectivity (22). In the structure, the substrate mimicked the extended conformation of an isopeptide bond between ubiquitin Gly76 and a lysine side chain (22). Based on this observation, we designed and generated fluorescent derivatives that mimic a substrate’s isopeptide bond and can largely be made recombinantly in easily scalable quantities. These were used to develop an FP assay system with wide applicability for measuring DUB activity and inhibition in low- and high-throughput format, which we will refer to as IsoMim assay. The assay will form a useful addition to available assay systems for inhibitor discovery and probing DUB mechanisms.

## Results

### Design principle and generation of novel deubiquitinase fluorescent reagents

**Figure 1:**
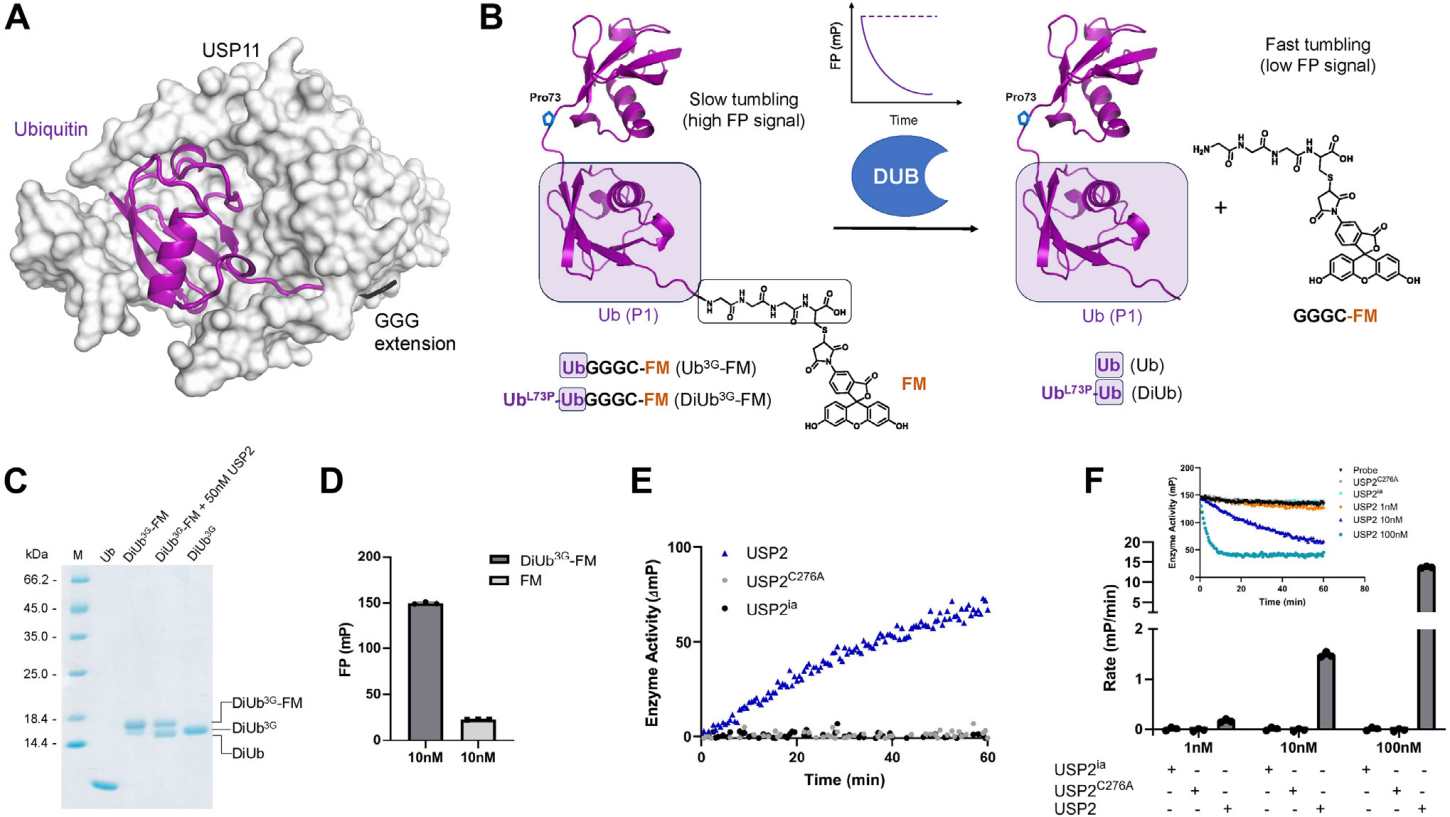
**Fluorescence polarization-based IsoMim assay principle and optimization.***A*, crystal structure of the catalytic core domain of USP11, depicted as a *grey* surface representation, in complex with Ub-GGG (in *purple* cartoon representation with the GGG extension colored in *black*; PDB code: 8OYP (22)) *B*, schematic depiction of the probe design and assay principle. Representation of the DiUb^3G^-FM substrate with the predicted Ub^L73P^-Ub structure (Alphafold (47)) in cartoon representation shown in *purple* and the C-terminal GGGC extension labeled with Fluorescein-5-maleimide (FM) shown as a chemical structure. The C-terminal ubiquitin moiety, the P1 position according to canonical protease nomenclature, is boxed and shaded in *purple* and forms the basis of a Ub^3G^-FM probe. The GGGC extension is highlighted by a *black-rimmed box*. Upon DUB cleavage of DiUb^3G^-FM, the rotational freedom of the liberated GGGC-FM moiety increases, leading to depolarization of the incident polarized light and a decrease in FP signal. *C*, SDS-PAGE gel analysis comparing mono-ubiquitin with the generated substrate DiUb^3G^-FM, in the absence and presence of 50 nM USP2 after 30 min incubation, and unlabeled DiUb^3G^. *D*, comparison of the FP signal from DiUb^3G^-FM (10 nM) with free fluorescein (10 nM) demonstrating the assay window. *E*, converted curves for 10 nM USP2, USP2^C276A^ and heat inactivated USP2 (USP^ia^) showing the FP change. *F*, representative progress curves for USP2, USP2^C276A^ and heat inactivated USP2 (USP^ia^) in comparison with the probe alone control (*top inset*). Rates of reaction at three different USP2 concentrations with 10 nM DiUb^3G^-FM (*bottom*). Data points represent the mean ± SD of three independent experiments.

Based on our earlier structural findings (22) that the engineered substrate Ub-GGG spans the active site in USP11 (Fig. 1*A*), mono- and linear di-ubiquitin-based fluorescent IsoMim probes were designed. The reagents harbor three additional glycines and a cysteine at the C-terminal ubiquitin moiety, allowing labeling with a fluorescent tag with a maleimide-fluorescent dye reagent (Figs. 1*B* and S1). DUB-mediated cleavage of such a fluorescent substrate is expected to liberate the fluorescent group and, as such, change the FP signal from this moiety (Fig. 1*B*). The di-ubiquitin reagent contains linear di-ubiquitin (M1-linkage) harboring a Leu73Pro mutation, which has been shown to prevent cleavage by DUBs (23). Ub-GGGC and Ub^L73P^-Ub-GGGC were generated recombinantly and conjugated with fluorescein-5-maleimide (FM) to yield Ub-GGGC-FM (abbreviated to Ub^3G^-FM) and Ub^L73P^-Ub-GGGC-FM (abbreviated to DiUb^3G^-FM), respectively. Typical yields obtained for the probes were 6 to 8 mg of pure protein per liter of culture. Labeling was conducted in 2-mg batches, and the yield from the labeling reaction after the removal of excess FM was around 65%. Incorporation of the fluorophore was confirmed by SDS-PAGE, which showed a clear gel shift to a higher molecular weight for both DiUb^3G^-FM and Ub^3G^-FM, compared to the non-labeled equivalent (Figs. 1*C* and S1). A faint lower molecular weight band was visible corresponding to the presence of a small amount of DiUb^3G^, estimated to be around 5% (Fig. 1*C*). Visualization by fluorescence scanning confirmed the presence of the fluorophore only after labeling (Fig. S1). Incubation of DiUb^3G^-FM with the catalytic domain of ubiquitin-specific protease 2 (USP2), a relatively promiscuous USP (24), generated a smaller fragment consistent with enzymatic cleavage of the di-ubiquitin substrate to Ub^L73P^-Ub and GGGC-FM (Fig. 1*C*). No further cleavage of Ub^L73P^-Ub to mono-ubiquitin was observed, confirming the validity of the Leu73Pro mutation and demonstrating the successful generation of the di-ubiquitin probe. Equally, Ub^3G^-FM was cleaved by USP2 (Fig. S1).

Next, the potential to use the DiUb^3G^-FM probe to monitor enzymatic cleavage by the digestive FP was investigated. To this end, it was first ascertained whether a suitable window for the digestive FP reaction existed. Comparison of the FP signal of free fluorescein with that of DiUb^3G^-FM in the absence of enzyme showed that at 10 nM the capability of DiUb^3G^-FM to depolarize the incident polarized light was significantly lower than that of free fluorescein suggesting a suitable dynamic range for detecting the liberated fluorescein moiety post DUB cleavage (Figs. 1*D* and S2). A similar pattern was observed for the titration of Ub^3G^-FM (Fig. S2). The difference in signal between free fluorescein and labelled probe was slightly higher for DiUb^3G^-FM than for Ub^3G^-FM, and therefore, subsequent enzyme activity assays in this study were developed using the DiUb^3G^ reagent.

All subsequent FP assays used were conducted at 10 nM diUb^3G^-FM. Enzyme activity was expressed as an increase in the level of depolarization. Progress curves of wild-type USP2, a catalytically inactive mutant USP2^C276A,^ and heat-inactivated USP2 (USP2^ia^), respectively, demonstrated cleavage of the probe, monitored by a decrease in the FP signal and an increase in depolarization (Fig. 1, *E* and *F*). Approximately tenfold increases in the reaction rates were observed at the corresponding increases in USP2 concentration (0.18 ± 0.03 mP/min for 1 nM USP2, 1.49 ± 0.05 mP/min for 10 nM USP2, and 13.68 ± 0.23 mP/min for 100 nM USP2). A catalytically inactive USP2^C276A^ mutant and heat-inactivated USP2 were not associated with any changes in signal (Fig. 1*F*). Thus, the assay is suitable to measure dose-dependent USP2-mediated ubiquitin deconjugation activity.

### The IsoMim assay can monitor the activity of different DUB family members

**Figure 2:**
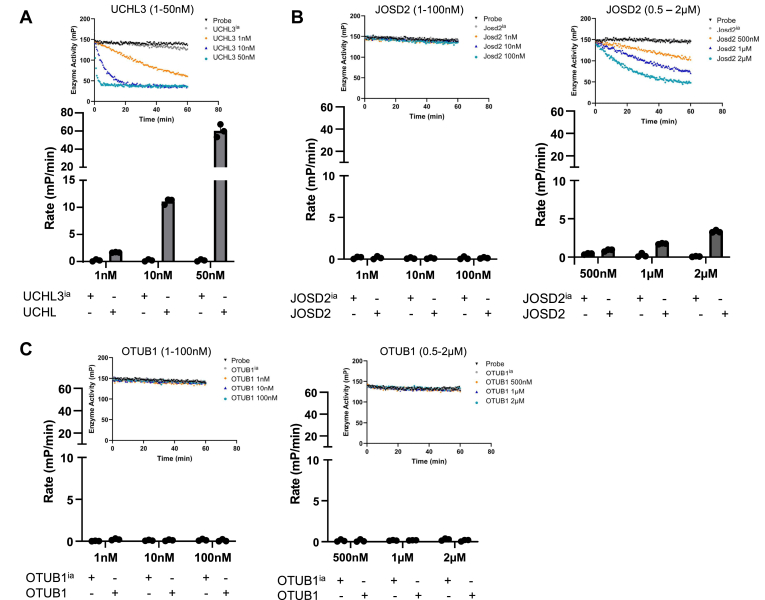
**Evaluation of assay applicability to DUBs from different sub-families.** Data are shown for (*A*) UCHL3, (*B*) JOSD2, and (*C*) OTUB1. Insets: representative progress curves are shown with highest (*teal*), medium (*blue*), and lowest (*orange*) enzyme concentrations as well as inactivated, denatured enzyme (*gray*) and the probe alone (*black*). Bars: Reaction rates at different enzyme concentrations were calculated from three independent experiments (*black dots*) and averages shown (error bar = SD). For OTUB1 and JOSD2, higher enzyme concentrations were used to capture low-level activity against the probe.

To evaluate the applicability and selectivity of the assay, we expressed, purified, and tested members of three other DUB families and compared these to USP2: UCHL3 as a member of the ubiquitin C-terminal hydrolase family, JOSD2 as a member of the Machado-Joseph domain-containing proteases, and OTUB1 as a member of the ovarian tumor protease family were selected (Figs. 2 and S3). The investigated DUBs are known to display different substrate scopes. In contrast to USP2, which is relatively promiscuous and able to cleave di-ubiquitin substrates of all linkage types (25), UCHL3 is known to be specific to small, disordered ubiquitinated substrates and cannot cleave any di-ubiquitin chains, regardless of the linkage type (26, 27). JOSD2 has weak activity towards several differently linked di-ubiquitin substrates (28) and has been reported to cleave Ub-Thr substrates (29), and OTUB1 is known to be highly specific for Lys48 linkages (30).

Three concentrations of UCHL3, JOSD2, and OTUB1 (1 nM, 10 nM, and 50 or 100 nM) were initially tested alongside the corresponding heat-denatured protease. UCHL3 was the most active among all the DUBs tested. At 50 nM, UCHL3 reached a cleavage rate of 60.1 ± 7.1 mP/min, which was around 5-fold higher than at 10 nM (11.1 ± 0.5 mP/min) and around 40-fold higher than at 1 nM concentration (1.67 ± 0.06 mP/min) (Fig. 2*A*). In contrast, at 100 nM concentration, JOSD2 and OTUB1 exhibited no measurable activity (Fig. 2, *B* and *C*). At 500 nM concentration, JOSD2 showed a rate of 0.88 ± 0.14 mP/min, which increased approximately 2-fold with each successive doubling of the enzyme concentration (1.76 ± 0.075 mP/min at 1 μM, and 3.34 ± 0.16 mP/min at 2 μM enzyme concentration) (Fig. 2*B*). No measurable activity was detected for OTUB1 at 2 μM concentration (Fig. 2*C*). The data revealed that using the IsoMim assay, differential activity among different DUB sub-families can be detected, consistent with their reported substrate scope, and suggest a wide applicability of the assay for many DUBs across different families.

### The IsoMim assay can be used in a competition format and can detect paralog-specific differences

**Figure 3:**
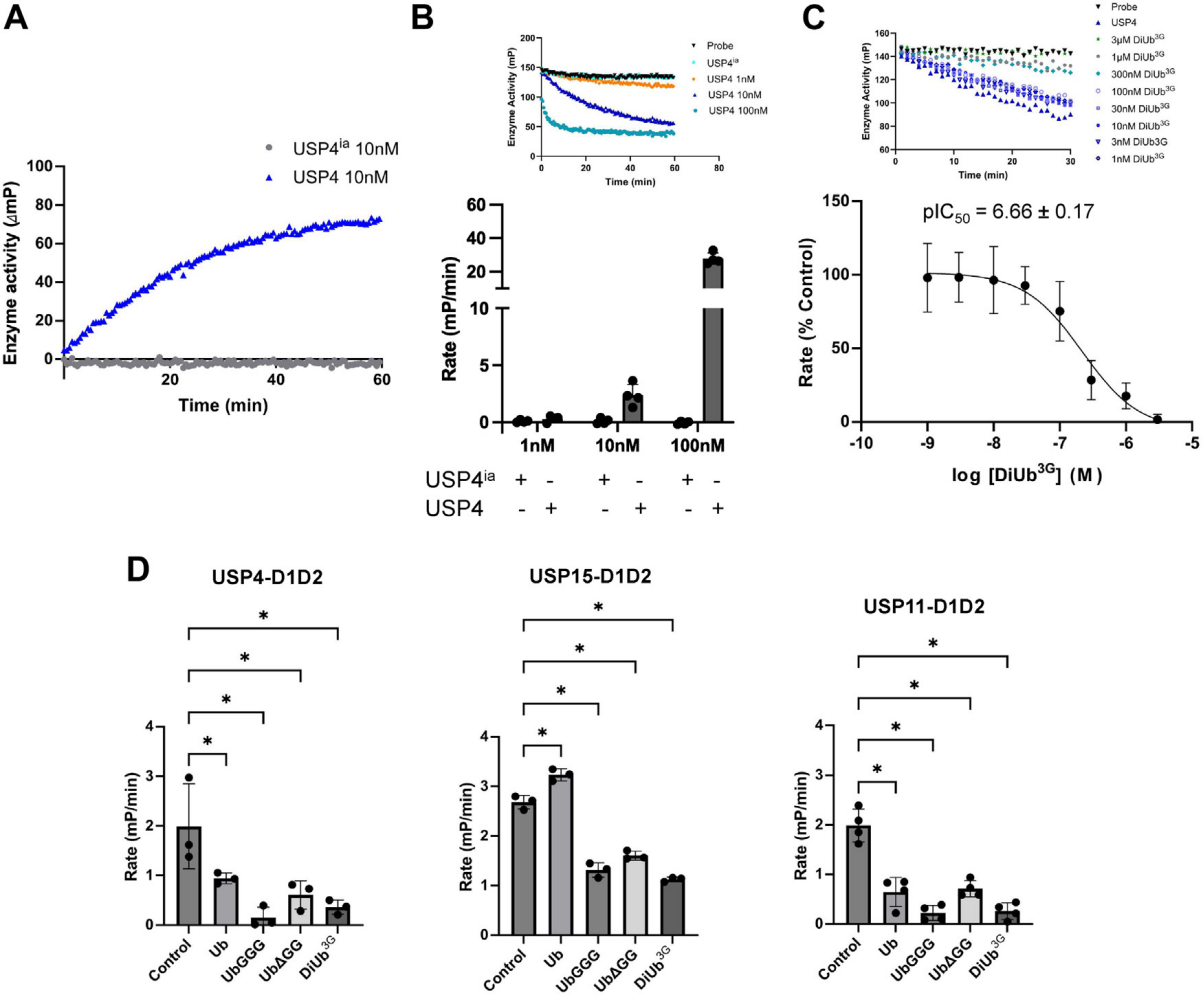
**Competition assays with an unlabeled probe and ubiquitin variants probing differences between paralogues USP4, USP15, and USP11**. *A*, progress curve of DiUb^3G^-FM cleavage by 10 nM USP4-D1D2 or heat-inactivated USP4-D1D2 over a period of 1 h. *B*, USP4-D1D2-mediated DiUb^3G^-FM cleavage rate at different USP4-D1D2 concentrations as indicated (mean ± SD, n = 4). The inset shows representative progress curves. *C*, concentration-dependent inhibition of USP4 activity by unlabeled DiUb^3G^ as a competitor (mean ± SD, n = 3). The inset shows representative progress curves. *D*, competition assays with product mono-ubiquitin (Ub), substrate Ub-GGG, the UbΔGG ubiquitin core, and unlabeled probe DiUb^3G^ for paralogues USP4, USP15, and USP11 (mean ± SD, n = 3–4). Control refers to the rate in the absence of a competitor. Statistical significance was determined using a one-way ANOVA, followed by Dunnett’s test for multiple comparisons. ∗ denotes significant difference between test and control group (*p* < 0.05). Specific *p* values (all *versus* control) were: for USP4, *p* = 0.039021 (Ub), *p* = 0.001141 (UbGGG), *p* = 0.008234 (UbΔGG), *p* = 0.002746 (DiUb^3G^). For USP11, *p* = 0.000003 (Ub), *p* < 0.000001 (UbGGG), *p* = 0.000005 (UbΔGG), *p* < 0.000001 (DiUb^3G^). For USP15, *p* = 0.000492 (Ub), *p* < 0.000001 (UbGGG), *p* = 0.000001 (UbΔGG), and *p* < 0.000001 (DiUb^3G^).

To further probe the scope of the assay, the catalytic core domain of USP4 (USP4-D1D2), which is less promiscuous compared to USP2, was tested (31). Progress curves over 1 hour were performed for three different USP4-D1D2 concentrations (Fig. 3, *A* and *B*). A useful window with an FP change of around 72.9 ± 2.9 mP was observed at 10 nM concentration of USP4-D1D2 (Fig. 3*A*). USP4-D1D2 cleaved the substrate in a concentration dependent manner with a mean cleavage rate of 0.29 ± 0.27 mP/min at 1 nM USP4-D1D2, 2.35 ± 0.97 mP/min at 10 nM USP4-D1D2 and 27.68 ± 3.41 mP/min at 100 nM USP4 -D1D2 (Fig. 3*B*). As for USP2, a USP4 concentration of 10 nM provided a good assay window and was used for all subsequent assays (Fig. 3*A*).

The established conditions were then employed for competition assays. The unlabeled probe DiUb^3G^ inhibited USP4-D1D2 activity with a pIC_50_ of 6.66 ± 0.17 (Fig. 3*C*). This demonstrated that inhibition by a substrate competitor can be successfully monitored. Comparisons with the catalytic core domains of paralogues USP15 (USP15-D1D2) (32) and USP11 (USP11-D1D2) (22) were then carried out. To this end, competition assays were conducted with the product mono-ubiquitin (Ub) and two ubiquitin variants: the engineered substrate mimetic Ub-GGG (22) and UbΔGG, equivalent to Ub (residues 1–74), and the unlabeled probe DiUb^3G^ to probe whether the assay is suitable for discerning paralogue-specific differences (Figs. 3*D* and S4). These showed that the reaction product mono-ubiquitin competes with the probe for USP4-D1D2, whereas it did not for USP15-D1D2. This is consistent with the known product inhibition of USP4 (31, 32). The substrates Ub-GGG and the unlabeled probe DiUb^3G^ similarly competed with the probe for USP15-D1D2, USP4-D1D2 and USP11-D1D2. On the other hand, UbΔGG, which lacks the C-terminal tail glycines required for conjugation to a target substrate, competed with the probe slightly better compared to mono-ubiquitin for USP15-D1D2 (Fig. 3*D*). The same differences were not observed for USP4-D1D2 and USP11-D1D2. USP11 displayed characteristics closer to USP4 than USP15 in the competition assays, consistent with the notion that it may have arisen from a duplication of the USP4-encoding region (33). The data show that the real-time monitoring of USP activity using the IsoMim assay method is suitable for competition assays and can provide insights into paralogue-specific differences.

### The IsoMim assay can assess the potency of a DUB inhibitor and is suitable for high-throughput screening

**Figure 4:**
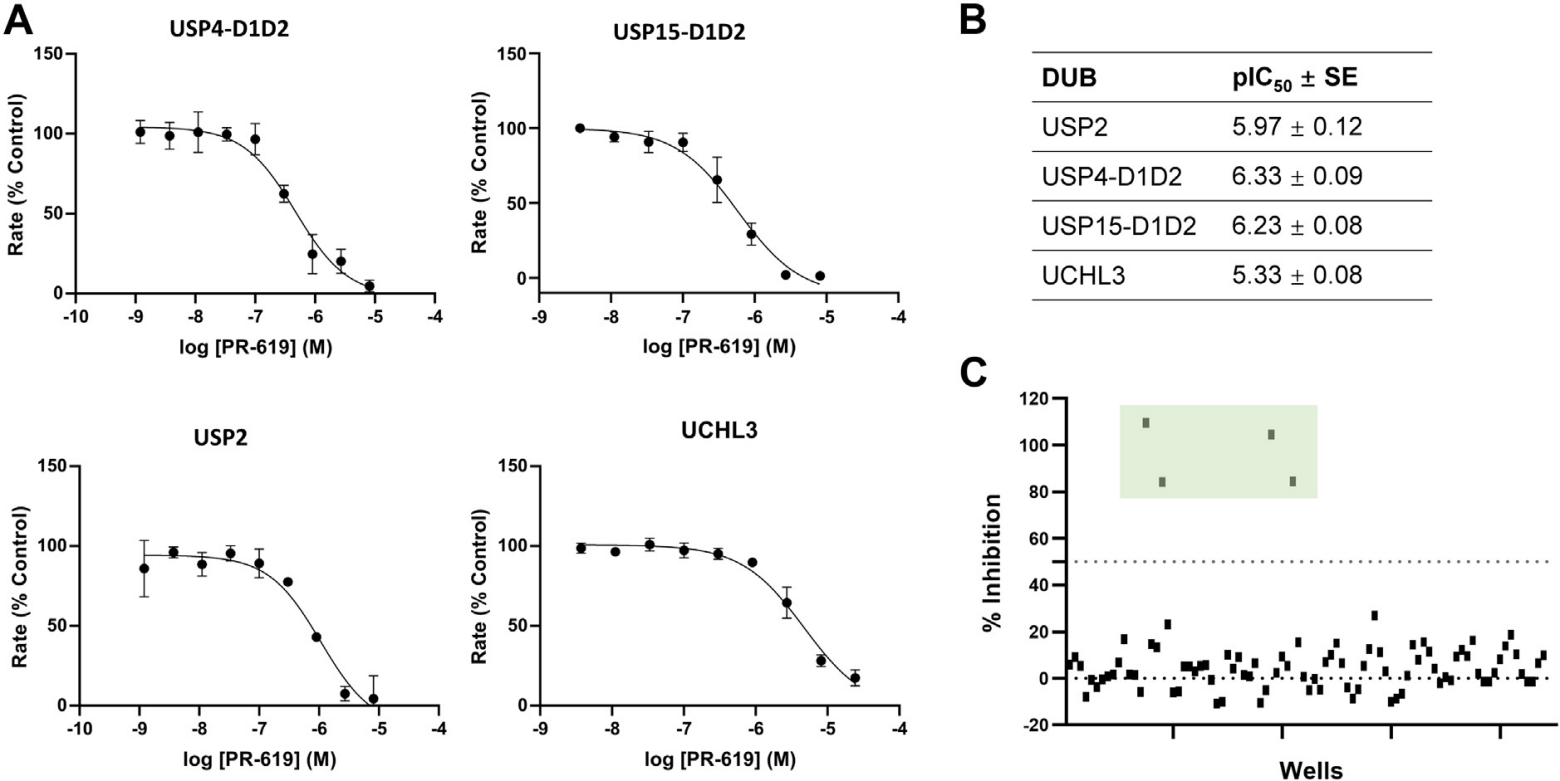
**Dose response curves of different DUBs and PR-619 as the inhibitor.***A*, dose–response curves for inhibition by pan-inhibitor PR-619 for the catalytic core domains of USP4, USP15, USP2 and UCHL3. Data were fitted using non-linear regression in GraphPad Prism, and data points show mean ± SD from three independent experiments. *B*, pIC50 values of PR-619 DUB inhibition (error = SE; n = 3). *C*, proof-of principle high-throughput screening results using USP4-D1D2 and PR-619 in a 384-well plate layout. The DiUb^3G^-FM reagent alone and selected wells together with USP4-D1D2 served as control groups, measured as quadruplicates on the plate as indicated in Fig. S5. PR-619 was added in random places in quadruplicate (plate layout displayed in Fig. S5). Highlighted in *light green* are the data from wells where USP4-D1D2 was incubated with PR-619. *Dashed lines* indicate 0% and 50% of inhibition.

To evaluate whether the assay method can be used for inhibitor identification and determination of potency, we measured the inhibitory potential of broad-range small molecule DUB inhibitor PR-619 (25). Dose response curves were acquired after pre-incubation with PR-619 for 30 min and concentration-dependent inhibition of USP4 activity was observed with a pIC_50_ of 6.33 ± 0.09 (Fig. 4, *A* and *B*). PR-619 also displayed dose-dependent inhibition of USP2, USP15-D1D2, and UCHL3 (Fig. 4*A*) with pIC_50_ values of 5.97 ± 0.12, 6.23 ± 0.08 and 5.33 ± 0.08, respectively (Fig. 4*B*). Although not directly comparable, the IC_50_ values determined using the IsoMim assay are broadly in line with reported EC_50_ values (25) demonstrating the reliability of the assay. A 384-well plate layout was then created, whereby all wells contained USP4-D1D2 and the DiUb^3G^-FM probe. PR-619 at 1 μM and 10 μM concentration was added as “pseudo hits” at random positions to assess the potential for hit identification in high-throughput screening. On the side of the plate, 10 nM of DiUb^3G^-FM in the presence and absence of 10 nM USP4-D1D2 was used as positive and negative control, respectively (Fig. S5). The Z′ factors determined from the experimental set-up were found to be in the range of 0.7 to 0.9, which is suitable for HT inhibitor screening. Analysis of the plate indicated that the wells containing PR-619 fell outside the 50% cut-off for inhibitor activity and these were thus identified as hit compounds (‘pseudo hits’). 1 μM PR-619 inhibited 80% of USP4-D1D2 activity and 10 μM PR-619 fully inhibited USP4-D1D2 activity in the assay (Fig. 4*C*). This demonstrates that the IsoMim assay is scalable and suitable for the identification and discrimination of DUB inhibitors in a high-throughput manner. To get insights into how the IsoMim assay performs against the widely used Ub-AMC fluorogenic assay (11), we measured a dose response curve for USP4-D1D2 and PR-619 in an analogous way and obtained a similar pIC_50_ value for the inhibition (Fig. S6*A*). Furthermore, the pseudo inhibitor screen was conducted in 384 well format that also showed comparable results to the IsoMim assay (Fig. S6, *B* and *C*). Higher concentrations of USP4-D1D2 and Ub-AMC reagent were required to obtain these results compared to the IsoMim assay. This showed that the IsoMim assay reliably measures activity and inhibition.

## Discussion

Fluorescence polarization assays are widely used for measuring enzymatic activity and in high-throughput screening, due to their robustness (the ability to circumvent various forms of assay interference) and fitness (various logistic limitations) (34). Here, we describe the development of IsoMim, a novel FP assay method for the measurement of deubiquitination activity that uses a recombinantly expressed substrate mimetic that is then fluorescently labelled. The data show that the extension of ubiquitin by additional glycines, although not a native isopeptide bond, sufficiently mimics the extended nature and length of the physiological isopeptide bond between the ubiquitin C-terminal glycine and a lysine side chain. The close alignment of the Ub-GGG core with the lysine side chain of a proximal ubiquitin moiety has also been shown in a crystal structure in complex with USP11 (22) when superimposed on a Lys6-linked diubiquitin–USP30 complex structure (35). The addition of a C-terminal cysteine in the reagent allows for labelling with any maleimide reagent of choice, making the assay suitable for a wide range of applications.

We chose to focus on a non-hydrolysable di-ubiquitin substrate, DiUb^3G^-FM, in this work due to its increased size compared to the analogous Ub^3G^-FM substrate but noted that the size difference between the Ub^3G^-FM substrate and the liberated fluorophore is also sufficient to detect DUB activity (Fig. S1). This is consistent with previously used semisynthetic isopeptide-linked substrates that are based on mono-ubiquitin (19, 20, 21). Despite recent advances in facilitating the generation of FP-based DUB substrates semi-synthetically (20), the finding that a glycine-based C-terminal extension is able to sufficiently mimic DUB substrates renders the generation of a minimal substrate more straightforward. The key benefit of this method is the ability to generate the assay reagents in high yields recombinantly, compared to other available assay methods, which require E1, E2, and E3 for chain assembly, intein chemistry or multi-step semi- or chemical syntheses not readily accessible to many laboratories (12, 20, 36, 37). The IsoMim method exhibits high sensitivity toward DUB activity by measuring the change in FP signal resulting from the molecular weight difference between di-ubiquitin (or mono-ubiquitin) and free GGGC-FM upon cleavage by a DUB. The current assay clearly measures enzyme activity, since heat inactivation or mutation of the active site cysteine abolishes enzyme activity. The excitation of fluorescein-5-maleimide at 495 nm lowers the risk of artifacts in screening due to auto-fluorescence of compounds compared to Ub-AMC. The way in which the fluorescent reagent was designed allows the possibility to incorporate alternative fluorophores to fluorescein, for example further red-shifted fluorophores such as TAMRA, which are generally preferred in HTS campaigns. Equally, the IsoMim principle should also be transferable to other ubiquitin-like modifiers such as SUMO or NEDD8, but this needs to be experimentally confirmed.

We successfully used the DiUb^3G^-FM reagent to detect and measure the activity of the catalytic core domains of USP2, USP4, USP15, and USP11. The selected USPs vary in their substrate scope, suggesting that the assay is suitable for measuring the activities of a wide variety of DUBs from the USP family with low reagent consumption. The assay was also suitable for UCHL3, which belongs to the UCH family, which usually displays a narrower substrate range (27, 38). JOSD2 activity was also measurable, albeit at higher concentrations, showing that the assay worked for a wide variety of DUBs across different families. We only found OTUB1, which is specific for Lys48-linked ubiquitin chains, to not cleave the engineered fluorescently labeled substrate, even at high concentrations. These findings are consistent with the generally weak activity of JOSD2 for di-ubiquitin substrates linked by isopeptide bonds *via* lysines (28) and the known highly specific nature of OTUB1 (30, 39). At the same time, this provides evidence that the current assay readout correlates with previously observed differential enzyme activity. We also showed that the assay can be used in the competition format by detecting differences in product and ubiquitin variant inhibition between close paralogues. The results suggested that USP4 is susceptible to product inhibition, as mono-ubiquitin competed well with the substrate, whereas this was not the case for USP15, in line with previous results (27, 31, 32). Mono-ubiquitin also competed well with the probe for USP11. The ubiquitin core lacking the two C-terminal tail glycines Gly75 and Gly76 (UbΔGG) displayed a similar competition behavior to mono-ubiquitin for USP4 and USP11. In contrast to mono-ubiquitin, UbΔGG unexpectedly competed with the probe for USP15. This may indicate initial contacts with USP15 engage the ubiquitin core rather than the C-terminal tail, although this will require further confirmation. Furthermore, the IsoMim assay detected inhibition of four selected DUBs by the known small-molecule broad-spectrum inhibitor, PR-619, and can be conducted at low substrate concentrations to determine dose–response curves. As the reagent can be produced in high yields in most laboratory settings in a few steps without the need for uncommon reagents, it is accessible to a wider research community and suitable for screening compound libraries.

Taken together, the IsoMim assay’s compatibility with members from different DUB families, real-time monitoring capabilities, and suitability for high-throughput screening have the potential to significantly aid research into a better understanding of DUB function, the identification of novel DUB inhibitors, and accelerate drug discovery campaigns.

## Experimental procedures

### Constructs and cloning

The gene coding for Ub^L73P^-Ub-GGGC was obtained by gene synthesis from GenScript and sub-cloned into expression vector pCDF-Duet. The construct consists of linear di-ubiquitin with a Leu73Pro mutation and an additional GGGC C-terminal extension. AGTATATTAGTTAAGTATAAGAAGGAGATATACATATGCAGATCTTCGTGAAGACCC/AGCGGTTTCTTTACCAGACTCGAGTCAACAACCGCCACCACC were the primers used for restriction free sub-cloning (40). Ub-GGGC was generated by PCR from a previously used Ub-GGG construct (22). The USP4-D1D2, USP15-D1D2 and USP11-D1D2 catalytic core constructs have previously been described (22, 32, 41). UCHL3 in pET-28a was a kind gift from Jorge Azevedo (42). OTUB1 in pET28a was kindly provided by David Komander (43). The USP2 catalytic domain expression construct as used for the structure determination of a covalent Ubiquitin-USP2 complex (PDB code: 2IBI) was a gift from Cheryl Arrowsmith (Addgene plasmid # 36894; http://n2t.net/addgene:36894; RRID: Addgene 36,894). The active site cysteine USP2^C276A^ mutation was generated by the QuickChange site directed mutagenesis kit (Qiagen). The JOSD2 (residues 12–186) gene was obtained by gene synthesis from GenScript and subcloned into pET21d using forward primer AATTTTGTTTAACTTTAAGAAGGAGATATACCATGACCGTGTACCACGAACGGC and reverse primer CTCAGTGGTGGTGGTGGTGGTGGTCTGTCCGCAGCCAGCTG. Mono-ubiquitin and Ub-GGG have previously been described (32). UbΔGG (residues 1–74) was generated by inserting a stop codon into the mono-ubiquitin construct.

### Protein expression and purification

The Ub^L73P^-Ub-GGGC (DiUb^3G^) and Ub-GGGC (Ub^3G^) constructs were expressed in LB broth medium with streptomycin and chloramphenicol in the *E. coli* BL21(DE3)-RIL codon plus strain. Cultures were grown at 37 °C to an OD_600_ of around 0.6 and further grown after induction by 0.5 mM isopropyl β-D-thiogalactoside (IPTG) for 3 h at 37 °C. Cells were harvested, lysed by sonication in buffer containing 20 mM ammonium acetate, pH 5.1, and centrifuged to remove any cell debris. The supernatant was treated with concentrated acetic acid to lower the pH to 4.5 ∼ 5.0. The resulting precipitate was removed by centrifugation, and the supernatant was treated with sodium hydroxide to adjust the pH to 5.1 before loading onto a cation exchange column (HiTrap SP FF, Cytiva). Protein was eluted by a linear ammonium acetate gradient. This was followed by size-exclusion chromatography on a Superdex75 16/60 column (GE Healthcare) using “reaction” buffer 20 mM Na-HEPES, 100 mM potassium acetate, pH 7.4 as running buffer. Relevant fractions containing pure DiUb^3G^ or Ub^3G^ were combined for fluorescein labeling after SDS-PAGE analysis. Ubiquitin variants were prepared and purified following the same procedure and stored in 20 mM Tris-Cl, pH 7.5, 150 mM NaCl, and 1% glycerol (GF buffer).

All DUB constructs were expressed in 2YT broth medium using *E*. *coli* BL21(DE3)-RIL codon plus strain. Cells were grown at 37 °C to an OD_600_ of around 0.6 and further grown after induction by 0.5 mM IPTG overnight at 20 °C. Cells were sonicated in Ni column buffer A: 50 mM Tris-Cl, pH 8, 300 mM NaCl, 20 mM imidazole, 10% glycerol, and then centrifuged to remove cell debris. Proteins were loaded onto a HiTrap chelating column pre-charged with nickel sulfate and eluted by an imidazole gradient. This was followed by size-exclusion chromatography using the GF buffer on a Superdex200 16/60 column (GE Healthcare). Fractions were analyzed by SDS-PAGE, and relevant fractions were combined, concentrated to 5 mg/ml, and stored at −80 °C.

### Generation and analysis of the probes

Fluorescein-5-maleimide (Sigma-Aldrich) was added to DiUb^3G^ or respective mono-ubiquitin aliquots (115 μM final concentration) in a 2:1 M ratio in buffer 20 mM Na-HEPES, 100 mM potassium acetate, pH 7.4 (reaction buffer). The solution was incubated overnight at 4 °C and protected from light. Excess fluorescein-5-maleimide was removed using a PD-10 column (Cytiva). The labelled probe was then subjected to a second size exclusion step for polishing using a Superdex 75 16/60 column with 20 mM Na-HEPES, 100 mM potassium acetate, pH 7.4 as the running buffer. Peak fractions corresponding to a dimer were discarded. The monomer fractions were pooled and concentrated and dialyzed in reaction buffer for dialysis overnight at 4 °C in the dark. Dialyzed samples were split into 10 μl aliquots and stored at −80 °C. The concentration of DiUb^3G^-FM was determined by measuring the absorbance at 495 nm using a nano-photometer using the following equation: [probe] = A_495nm_/(E x L), whereby an extinction coefficient of E = 68,000 M^−1^ cm^−1^ for fluorescein-5-maleimide was used and the path length L equals 1 cm (44). SDS-PAGE gel visualization on a fluorescence scanner (Odyssey XF Imager) and subsequently stained with Coomassie brilliant blue was used to confirm the incorporation of the fluorophore.

### FP assay window and DUB activity assay

All FP measurements were carried out on a PerkinElmer plate reader in black 384-well microplates (Corning) using 480 nm excitation and 535 nm emission filters and parallel and perpendicular detectors. FP values were calculated using the following equation: mP value for FP measurement = 1000 ∗ (S − G ∗ P)/(S + G ∗ P) where S is the parallel emission intensity, P is the perpendicular emission intensity, and G is the gain factor (45).

For determination of the assay window, a tenfold serial dilution series ranging from 100 nM to 1 pM Ub^3G^-FM or DiUb^3G^-FM probes or free fluorescein-5-maleimide were prepared in PBS buffer in a total volume of 50 μl at 22 °C. Each concentration was tested in triplicate and FP values were obtained as described above.

For DUB activity, assays were performed in 50 μl PBS buffer with 1 mM DTT and 0.02% Tween 20 at a final concentration of 10 nM of DiUb^3G^-FM. Heat-inactivated DUB negative controls were prepared by heating samples at 95 °C for 20 min prior to the addition of the probe. Assays were started by the addition of DiUb^3G^-FM and FP measurement proceeded with readings taken every 30 s for 120 repeats. Raw polarization values were converted to enzyme activity values by subtracting the probe- (DiUb^3G^-FM) only control and taking the absolute value (|mPprobe + enzyme – mPprobe|), reflecting the extent of depolarization. Rates were calculated from the linear phase of the reaction. Data were analyzed using GraphPad Prism. Each data point represents the mean ± standard deviation of three independent experiments.

### Competition assays

Each reaction contained 10 nM DiUb^3G^-FM and 10 nM of selected DUBs in PBS buffer with 1 mM DTT and 0.02% Tween 20. Competitors DiUb^3G^, mono-ubiquitin, UbΔGG, Ub-GGG or pan-DUB inhibitor PR-619 were first diluted to 10 times the desired assay concentration and then pre-incubated with the respective DUB at 22 °C for 30 min. Competition assays were carried out in the presence of excess DiUb^3G^, mono-ubiquitin, UbΔGG, and Ub-GGG in the range of (1 μM for USP4 and USP11, 3 μM for USP15). The reactions were initiated by adding 15 μl of substrate (33.3 nM) to achieve a final concentration of 10 nM in a total volume of 50 μl. Readings were taken every 30 s for 120 repeats. Rates were calculated from the linear phase of the reaction, and the pIC_50_ values, the concentration required for 50% inhibition, were calculated in GraphPad Prism using non-linear regression analysis. Data from three independent experiments were combined into a single graph, normalized to percentage activity, and presented as percentage values. Error bars on the data points represent standard deviation (SD). The calculated pIC_50_ values are reported with standard error (SE) of the regression analysis. Statistical significance was determined using a one-way ANOVA, followed by Dunnett’s test for multiple comparisons.

### Proof-of-concept high-throughput screening test using USP4-D1D2

15 μl of 33.3 nM USP4-D1D2 was added to a 384-well assay plate and incubated for 30 min at 22 °C. Some wells contained either one or 10 μM PR-619, as shown in Fig. S5. The reaction was started by the addition of 15 μl (33.3 nM) DiUb^3G^-FM probe to make up a total assay volume of 50 μl, and FP signal readings were measured after 20 min. The DiUb^3G^-FM reagent alone or together with USP4-D1D2 was used as a positive and negative control, respectively. The Z′ factor was calculated using the following equation: Z’ = 1 - (3SD_p_ + 3SD_n_)/|μ_p_ - μ_n_|; whereby SD_p_ and SD_n_ are the standard deviation of the positive and negative control, μ_p_ and μ_n_ are the mean value of the positive and negative control, respectively (46). The percentage of inhibition was calculated using the following equation: % Inhibition = 100 × (mPn – mPc)/(mPn – mPp); whereby mPn represents FP values of the negative controls, mPp for positive controls, and mPc for compound samples.

### USP4-D1D2 activity measurements using Ub-AMC as substrate

Reactions were carried out in 30 μl of 50 mM Tris-Cl pH 7.5, 150 mM NaCl, 1 mM DTT in 384-well black plates at final concentrations of 1 μM Ub-AMC (Ubiquigent Ltd) and 50 nM USP4-D1D2. PR-619 concentrations for the dose–response curve ranged from 81 μM, to 37 nM. Samples were incubated at 22 °C for 30 min, and reactions were initiated by adding 3 μl of the Ub-AMC stock solution. Wells were read with an Envision plate reader at 360 nm excitation and 460 nm emission, and data points recorded every minute for 50 min. Data were analyzed using GraphPad Prism. Rates were calculated from the linear phase of the reaction. Nonlinear regression analysis was used to derive the pIC_50_ value from the dose response curve. Data from three independent experiments were combined and presented as percentage values. For the inhibitor discovery pilot screen 10 μl of 150 nM USP4-D1D2 were added to a 384-well assay plate and incubated for 30 min at 22 °C. Some wells contained either one or 10 μM PR-619 as shown in Fig. S6*C*. The reaction was started by the addition of 3 μl of the 10 μM Ub-AMC stock solution to make up a total assay volume of 30 μl. The fluorescence signal at 460 nm was measured at the 30-min time point, which served as the endpoint for data analysis. Ub-AMC alone or together with USP4-D1D2 was used as positive and negative controls, respectively.

## Data availability

The data that support the findings of this study are included in the article or are available from the corresponding authors upon request.

## Supporting information

This article contains supporting information.

## Conflict of interest

The authors declare that they have no conflicts of interest with the contents of this article.
