# Supplementary material for: A versatile fluorescence polarization-based deubiquitination assay using an isopeptide bond substrate mimetic (IsoMim)
Source: J Biol Chem. 2025 Jun 6;301(7):110342. doi: 10.1016/j.jbc.2025.110342 (PMC12273560; doi:10.1016/j.jbc.2025.110342)
Supplement: Supporting Information [file mmc1.pdf]

A versatile fluorescence polarization based deubiquitination assay using an isopeptide bond substrate mimetic (IsoMim)

Jiatong Zhang [1], Jed Allen [1], Stephanie J. Ward [1,2], Lodewijk V. Dekker* Ingrid Dreveny*

Biodiscovery Institute, School of Pharmacy, University of Nottingham, Nottingham NG7 2RD, United Kingdom.

* To whom correspondence should be addressed: Lodewijk Dekker lodewijk.dekker@nottingham.ac.uk or Ingrid Dreveny ingrid.dreveny@nottingham.ac.uk Biodiscovery Institute, School of Pharmacy, University of Nottingham, Nottingham NG7 2RD, UK

Content:

Figure S1: SDS-PAGE gel confirming probe labelling

Figure S2: Evaluation of the assay window at different probe concentrations

Figure S3: Proteins used for the IsoMim assay development

Figure S4: Competition assays representative experiments

Figure S5: High-throughput pilot assay using USP4-D1D2 and pan-DUB inhibitor PR-619

Figure S6: USP4-D1D2 inhibition assays using the fluorogenic substrate Ubiquitin-AMC

Supporting Fig. 1

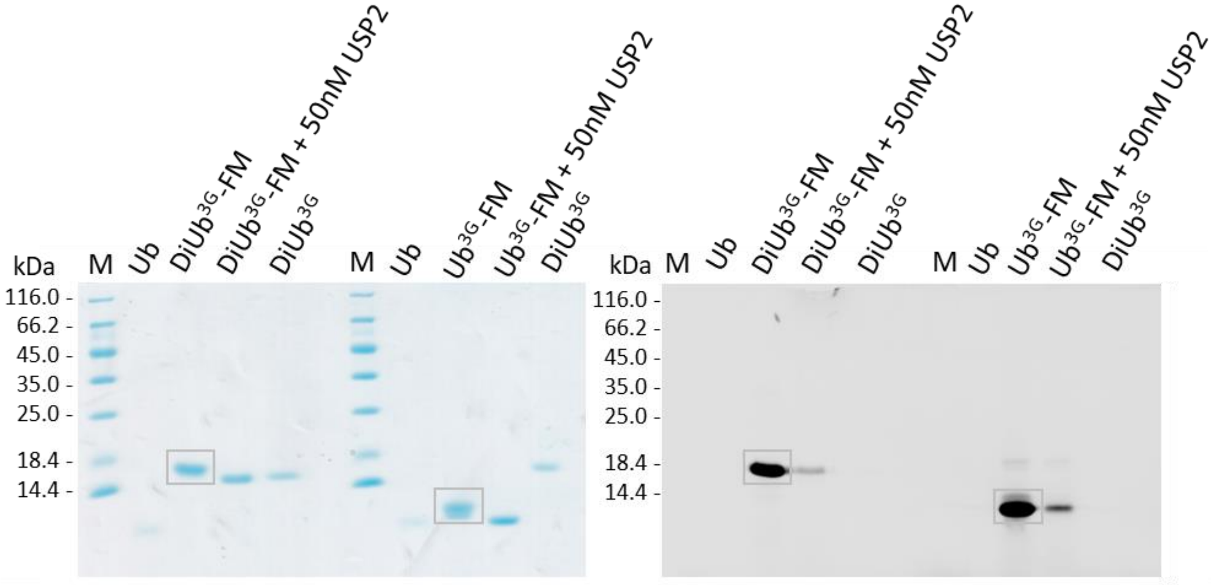

Figure S1: SDS-PAGE gel confirming probe labelling. SDS-PAGE gel analysis comparing mono-ubiquitin with the generated substrates DiUb^{3G}-FM and Ub^{3G}-FM, in the absence and presence of 50 nM USP2 after 45 min incubation, and unlabeled probe as indicated. The same gel was visualized using a fluorescence scanner (right) and subsequently stained with Coomassie Brilliant blue (left). Only the lanes with the fluorescein labelled IsoMim assay reagents are visible under the fluorescence scanner (boxed in gray) and the intensity of the bands upon USP2 cleavage decreases.

Supporting Fig. 2

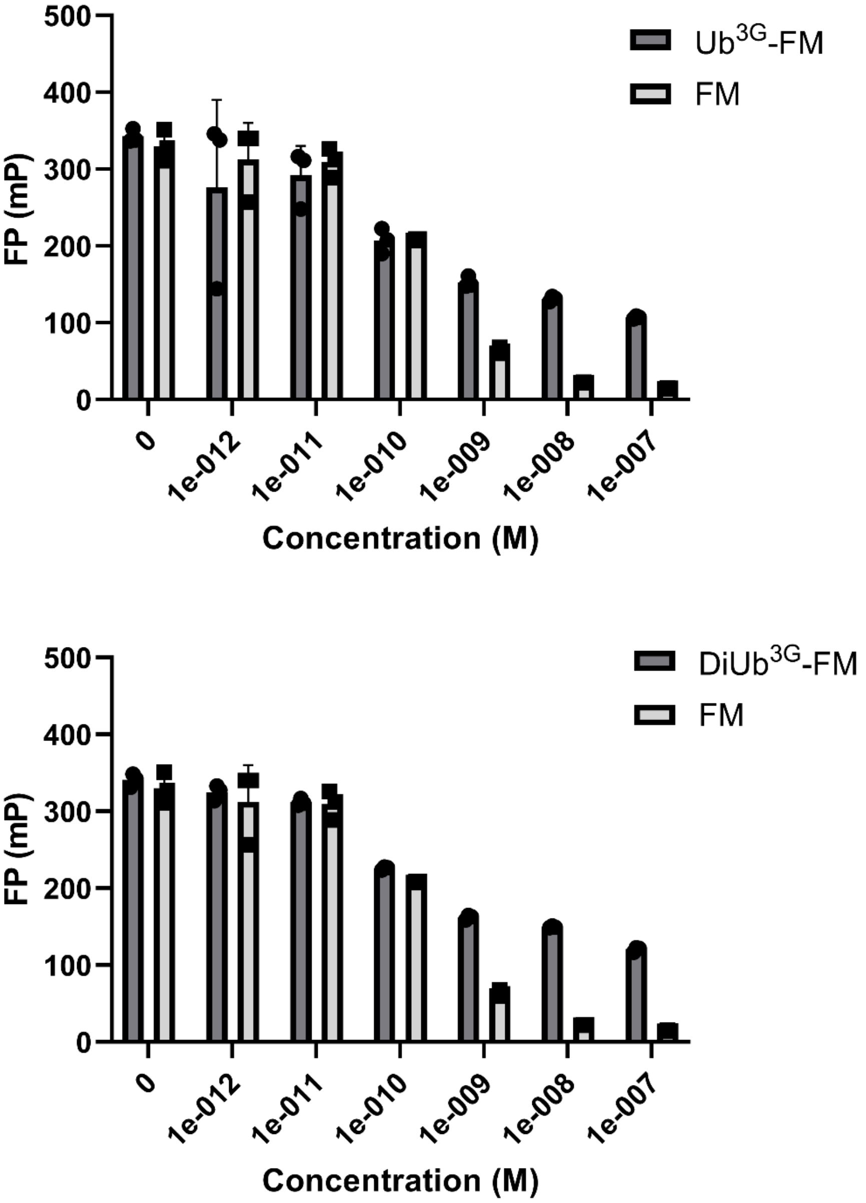

Figure S2: Evaluation of the assay window at different probe concentrations. The FP signal between free fluorescein (FM) and Ub^{3G}-FM (top) and $DiUb^{3G}$-FM (bottom) probes were measured at different concentrations to determine suitable concentrations for measuring a reliable signal and evaluating the assay window.

Supporting Fig. 3

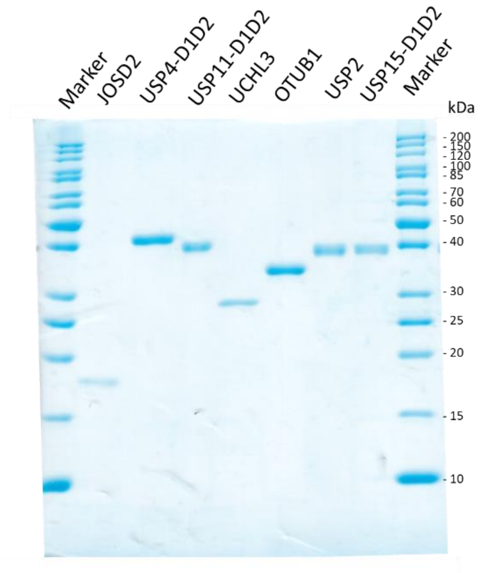

Figure S3: Proteins used for the IsoMim assay development. SDS-PAGE gel loaded with purified protein samples used for the assay development as indicated above the lanes from left to right: JOSD2, USP4-D1D2, USP11-D1D2, UCHL3, OTUB1, USP2 and USP15-D1D2.

Supporting Fig. 4

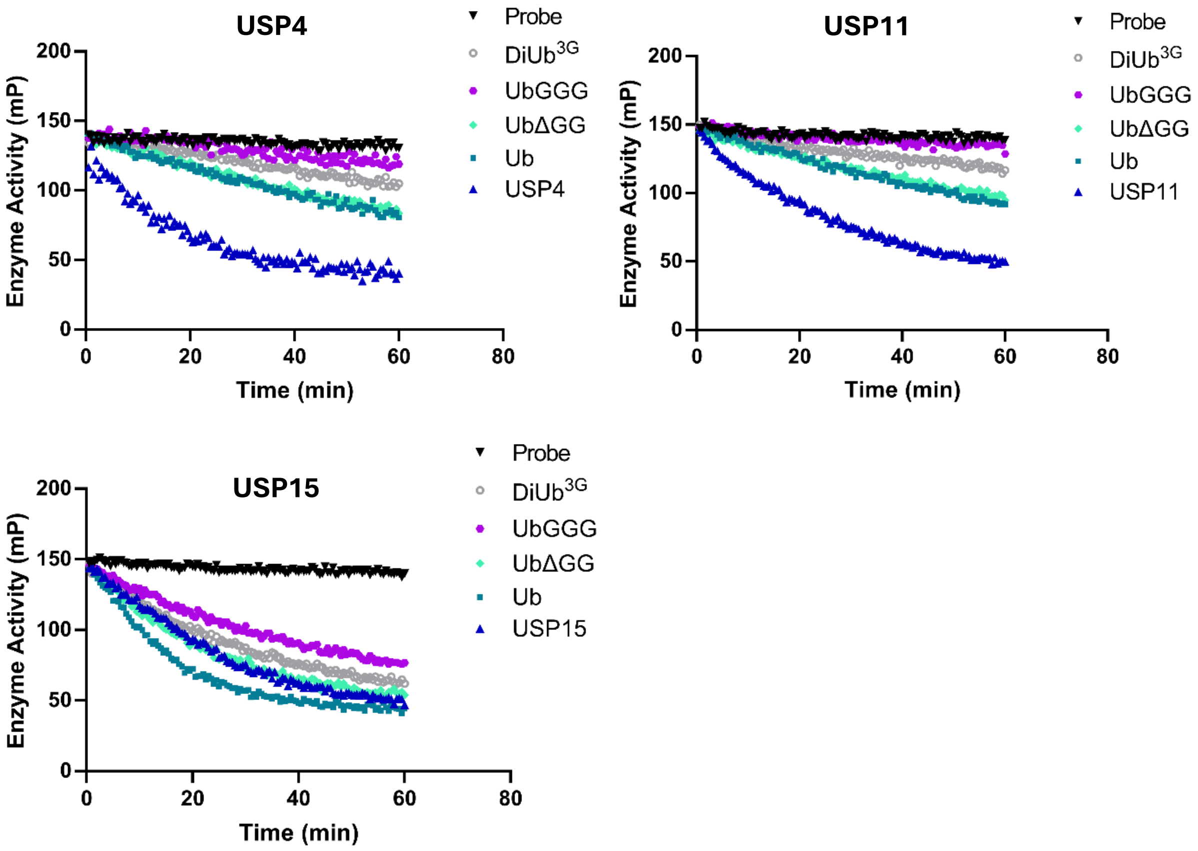

Figure S4: Competition assays representative experiments. Representative progress curves of competition assays with product mono-ubiquitin (Ub), substrate Ub-GGG, the UbΔGG ubiquitin core and unlabeled probe DiUb^{3G} for paralogues USP4, USP15 and USP11.

Supporting Fig. 5

	1	2	3	4	5	6	7	8	9	10	11	12	13	14	15	16	17	18	19	20	21	22	23	24
A	Control																							
B	Control																							
C	Control					1 µM PR-619						1 µM PR-619												
D	Control																							
E																								Control
F																								Control
G																								Control
H																								Control
I	Control																							
J	Control																							
K	Control									10 µM PR-619														
L	Control																							
M				10 µM PR-619																				Control
N																								Control
O																								Control
P																								Control

	1	2	3	4	5	6	7	8	9	10	11	12	13	14	15	16	17	18	19	20	21	22	23	24
A	149.2	93.7	83.2	89.9	82.3	93.4	100.5	97.9	93.9	96.2	78.1	85	86.8	84.1	96.7	101.5	96.2	88.9	86.1	106	83.3	93.7	94.6	96.4
B	148.8	83.6	99	87.6	89.3	90.9	94.4	72.7	94	80.3	93.6	85.9	100.7	87.4	102.6	88.6	128	85.3	104.8	91	95.7	94.3	98.1	93.5
C	148.2	99	87.8	87.2	84.6	144.7	132.3	88.2	92.2	89.9	80.3	141.4	142.7	96.9	99.2	97.8	93.4	96	92.5	89.9	96.4	109.6	93.9	78.2
D	155.2	87.1	94.4	93.7	96.4	140.6	144.4	89.9	89	88.1	88.8	141	137.5	88.5	99.1	87.3	94.8	93.6	102.7	82.5	100.6	95	94.8	93.4
E	86.8	93.6	86.9	107.3	89.5	135.1	91	89.6	90.6	101.5	88.3	98	94.4	98	89.2	87	90.1	93.8	101.7	106.5	92.9	94	96.5	149.3
F	83.2	83.2	94.9	89.6	101.7	92.8	85.9	78.2	84.3	85.3	86.2	90.2	102	86.9	87.5	85.8	89.7	86.4	92.5	84.8	102.4	90.4	90	152.4
G	81.9	73.4	84.5	98.7	87.2	83.7	82.9	68.5	83.7	76.8	82.3	90.6	82.7	76.3	86.9	79.9	79.2	100	82.5	94.9	87	98.6	80.7	150.5
H	77	83.7	82.4	83.7	79.3	80.5	82.1	82.3	82.6	77.5	81.2	87.4	86	81.7	89.6	77	82.7	87.4	85.3	91.3	76.7	89.1	81.1	152.3
I	155.9	84	90.3	93.2	85.7	75.9	90.5	83.5	71.5	80.8	84.1	82.4	80	84.8	74.1	83.8	86.4	85.5	76.3	87.4	89	91.8	75.5	78.7
J	146.5	83.5	84.9	85.3	84.3	74.7	88.9	79.1	84.7	84.8	81.5	84.7	84.4	75.9	87.3	77.1	75	90.9	86.6	81.2	83.6	82	91.7	100.6
K	153.7	84.4	87	85.5	74.3	90.6	85	84.6	84.6	153.8	147.6	87.1	80.5	92.2	73.2	85.5	79.1	90.2	75.1	83.8	80.9	84.6	80.4	81.7
L	153.5	78.7	84.8	81.3	88.6	83.7	98.7	86.8	114.8	155.9	157.3	80.4	95.8	82.7	84.3	82.7	80.6	90.2	90.3	85.3	91.1	84.5	91.8	84
M	85.4	84.5	87.1	161.8	154	88.9	91.8	85.2	94	85.1	80.4	83.4	93	78	90.5	78.6	84.4	86.6	88.5	88.2	86.8	91.4	96.4	153.6
N	85.5	82.9	89.1	152.5	159.4	80	97.3	83.3	92.8	92	92.9	90.2	65.5	94.1	95.7	92.5	91.9	81.4	85.6	84.4	91.5	90.4	83	137.9
O	88.7	81.9	93.6	92.6	100.4	83.9	99.1	92	95.2	89	96	88.8	99.5	98.6	99.3	93.5	99.1	92.5	96.2	88.8	95.4	94.6	97.4	151
P	84	81.4	90.3	93.8	95.8	83.8	85.8	87.2	93.6	85.7	98.3	86.9	87.4	92.9	86.6	97.1	92.2	91	88.8	84.2	97.6	93.4	84.4	154

Figure S5: High-throughput pilot assay using USP4-D1D2 and pan-DUB inhibitor PR-619 as model DUB and inhibitor, respectively. Plate layout (top) and plate reader measurements (bottom) as taken from a 384 well assay plate. In the plate layout wells depicted in white contained 10 nM DiUb^{3G}-FM assay reagent and 10 nM USP4-D1D2, wells depicted in blue contained probe only (positive controls) and wells colored in red contained USP4-D1D2 and PR-619 at 1 or 10 µM concentration as indicated. Shown below is a corresponding heat map of the raw data measurements, with higher FP values represented in deeper shades of red.

Supporting Fig. 6

A

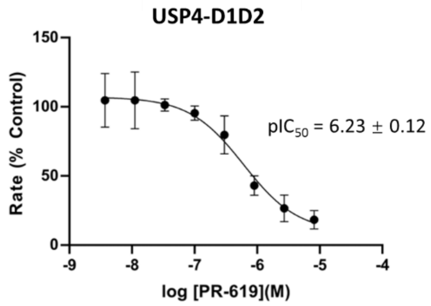

B

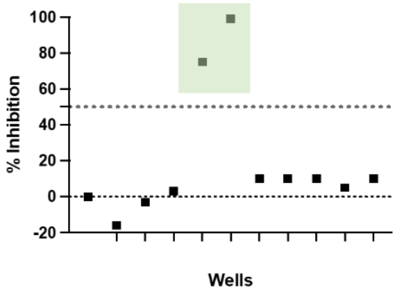

C

	1	2	3	4	5	6	7	8	9	10	11	12	13	14	15	16	17	18	19	20	21	22	23	24
A	Control							1 μM PR-619								10 μM PR-619								
B																								

	1	2	3	4	5	6	7	8	9	10	11	12	13	14	15	16	17	18	19	20	21	22	23	24
A	782	879	1743	2121	1926	1985	1875	1148	1083	2234	1957	2058	1907	2063	1836	940	743	1807	1825	1830	1946	1754	1726	1813
B	881	749	1899	1904	2013	2445	1851	1151	991	1848	1659	1859	1684	1805	1842	829	819	1763	1703	1868	1866	1655	1754	1924

Figure S6: USP4-D1D2 inhibition assays using the fluorogenic substrate Ubiquitin-AMC. ***A*** Dose response curve and derived pIC50 value for inhibition by pan-inhibitor PR-619 for the catalytic core domain of USP4. Data were fitted using non-linear regression in GraphPad Prism, and pIC_{50} value is reported as mean ± SE from three independent experiments (error = SE; n = 3). ***B*** Ub-AMC reagent alone and selected wells together with USP4-D1D2 served as control groups, measured as quadruplicates on the plate as indicated. PR-619 was added in random places in quadruplicate as shown in the plate layout (***C***). Highlighted in light green are the data from wells where USP4-D1D2 was incubated with PR-619. Dashed lines indicate 0% and 50% of inhibition.
